# Randomized control trial of advanced cancer patients at a private hospital in Kenya and the impact of dignity therapy on quality of life

**DOI:** 10.1186/s12904-020-00614-0

**Published:** 2020-07-23

**Authors:** John Weru, Miriam Gatehi, Alice Musibi

**Affiliations:** 1 Palliative care, AKUHN, Nairobi, Kenya; 2 MMED INT. Med, Nairobi, Kenya; 3 Oncology, Kenyatta National Hospital, Nairobi, Kenya

**Keywords:** Dignity therapy, Palliative care, Quality of life, Advanced cancer patients, Edmonton symptoms and assessment scale

## Abstract

**Background:**

Palliative care is a modality of treatment that addresses physical, psychological and spiritual symptoms. Dignity therapy, a form of psychotherapy, was developed by Professor Harvey Chochinov, MD in 2005.The aim of the study was to assess the effect of one session of dignity therapy on quality of life in advanced cancer patients.

**Methods:**

This was a randomized control trial of 144 patients (72 in each arm) randomized into group 1 (intervention arm) and group 2 (control arm). Baseline ESAS scores were determined in both arms following which group 1 received Dignity therapy while Group 2 received usual care only. Data collected was presented as printed (Legacy) documents to group 1 participants. These documents were a summary of previous discussions held. Post intervention ESAS scores were obtained in both groups after 6 weeks. Analysis was based on the intention to treat principle and descriptive statistics computed. The main outcome was symptom distress scores on the ESAS (summated out of 100 and symptom specific scores out of 10). The student T-test was used to test for difference in ESAS scores at follow up and graphs were computed for common cancers and comorbidities.

**Results:**

Of the 144 (72 patients in each arm) patients randomized, 70%were female while 30% were male with a mean age of 50 years. At 6 weeks, 11 patients were lost to follow up, seven died and 126 completed the study. The commonly encountered cancers were gastrointestinal cancers (43%, *p* = 0.29), breast cancer (27.27% *p* = 0.71) and gynaecologic cancers (23% *p* = 0.35). Majority of the patients i.e. 64.3% had no comorbidities.

The primary analysis results showed higher scores for the DT group (change in mean = 1.57) compared to the UC group (change in mean = − 0.74) yielding a non-statistically significant difference in change scores of 1.44 (*p* = 0.670; 95% CI − 5.20 to 8.06). After adjusting for baseline scores, the mean (summated) symptom distress score was not significant (GLM *p* = 0.78). Dignity therapy group showed a trend towards statistical improvement in anxiety (*p* = 0.059). The largest effects seen were in improvement of appetite, lower anxiety and improved wellbeing (Cohen effect size 0.3, 0.5 and 0.31 respectively).

**Conclusion:**

Dignity therapy showed no statistical improvement in overall quality of life. Symptom improvement was seen in anxiety and this was a trend towards statistical significance (*p* = 0.059).

**Trial registration:**

Trial registration number PACTR201604001447244 retrospectively registered with Pan African Clinical trials on 28th January 2016.

## Background

Cancer by definition is a disease characterized by the abnormal proliferation of DNA secondary to somatic mutations which could be primary genetic with environmental influences [1]. Thirty percent of cancers are curable if detected early; 30 % are treatable with prolonged survival if detected early and another 30 % can be provided with adequate symptom management and palliative care [2].

In Kenya, cancer is the third highest cause of mortality (7% of deaths) per year, first in line being infectious diseases e.g. HIV followed closely by cardiovascular diseases. Estimates in the country have shown that new cases are up to 39,000 per year with more than 27,000 deaths per year of whom majority are less than 70 years of age [3]. Most cancer patients present late to health facilities due to various factors which include both psychosocial and economic factors [4]. Treatment dynamics in these patients have moved from purely curative to palliative care models primarily to improve quality of life.

Targeted psychotherapy namely dignity therapy has been utilized in this study [5–8]. Dignity therapy is by definition a “unique, individualized psychotherapy that allows patient to revisit their lives and find meaningful events, persons and experiences hence maintaining their dignity” [5]. Studies have illustrated good outcomes in lowering depression, anxiety and grief in these patients in addition to numerous other benefits [5, 6, 9].

The inventor of Dignity therapy is a well-known professor of psychiatry known as Dr. Harvey Max Chochinov [5, 9, 10] who developed it as a means of assisting people dealing with the imminent end of their lives. The therapy includes 10 core questions [5, 6, 9] that address the most important accomplishments, lessons in life, hopes and dreams for loved ones and many more. (See Additional file 1). The trained therapist leads the patient through a session with open-ended questions usually between 30 and 60 min after rapport is established. The patients are free to discuss their lives events freely and the conversation is recorded, transcribed, edited and returned within a few days. The therapy creates something that will transcend the patient’s death and extend his or her influence across time.

There have been numerous trials with some efficacy studies pioneered by Chochinov himself [2, 5, 9] who conducted the largest known study to date in 2011.Other smaller studies by Hall et al. [11–14], Juliao et al. [5, 12, 15–17] etc. have been conducted with the most recent one being in 2017 [18–20].

Notably, the largest study [9] which included 441 patients divided in 3 groups, was among the few powered to detect small or moderate outcomes in the study. In this study, there were no statistically significant differences in primary outcomes in patients receiving DT as compared to the other two groups. In the newer studies from 2016 to 2018, most had improvement in primary outcomes [12, 17].

None of the studies addressed Quality of life as a primary outcome which the basis of this study.

The tool used in evaluation of quality of life outcomes was the revised Edmonton symptom assessment scale (see Additional file 1). This tool has been validated in previous studies [21–25] and was in use in AKUHN (Agakhan University Hospital, Kenya) at the time of the study.

## Methods

This study adheres to CONSORT guidelines.

The Primary Objective of the study was to compare dignity therapy vs usual care on quality of life of advanced cancer patients undergoing palliative care. Quality of life was measured using the Edmonton symptom scale (ESAS).

This was a parallel design randomized control trial set at the Aga Khan University hospital, which is a private referral hospital located in Kenya’s capital city Nairobi. Patients were recruited from the inpatient hospital setting and outpatient oncology clinics, which included surgical oncology, medical oncology and radiation oncology. The study population included all English-speaking adults aged between 18 and 65 years with advanced cancers (stage 3 and 4) at Aga Khan University hospital Nairobi.

All patients with any acute illness that was easily treatable/reversible (in addition to the underlying cancer) were excluded since they could skew the results especially on recovery.

This study was a superiority trial with a 1:1 parallel design. The power was set at 80% with a set *p*-value of 0.05. The sample size was 144, 72 per arm.

Recruitment was conducted by the principal investigator who was assisted by triage nurses allocated to the respective clinics. Informed consent was obtained from each patient and patients who were cognitive but could not sign due to disability or otherwise used thumb prints. Simple randomization was carried out using a computer generated randomization list with the help of a statistician into two groups. The first was the intervention arm where an ESAS form was filled prior to dignity therapy (and their usual care). The second group i.e. the control group had the ESAS filled before they received their usual care.

There was single blinding i.e. the patients only. The outcome assessors (counsellors) and the primary investigator were not blinded.

Trained counsellors/primary investigator through oral interviews, which were tape-recorded and/or transcribed on paper, administered dignity therapy. This was a single session; no additional sessions were given during the course of the study. This is mainly due to the fact that a pre-test had been done in the quality controlled sessions involving the counsellors and had been found to be appropriate for the study. The sessions were conducted in a private setting i.e. room in clinic or bedside by the provider who delivered these services at least once to the patients in the intervention arm. The counsellors first introduced themselves, established rapport and administered the pre-intervention tool i.e. ESAS within an average of 10 min. This was before the actual intervention involving Dignity therapy.

Dignity therapy(intervention) sessions were then conducted and since majority of the patients were stable, time taken was on average 30–60 min according to previous studies [5, 6, 9, 26]. The counsellors were trained 3 months prior to the study with practice sessions involving live patients. The training involved a brief of dignity therapy and videos of professional dignity therapists conducting the interviews. Quality control measures were undertaken by using the standardized template to conduct interviews i.e. involving Dignity therapy. This was the printed version of Chochinov’s protocol. These sessions were tape recorded (before the start of the study and after the study was conducted), selected at random and reviewed by the primary investigator to ensure standardization was occurring.

### Follow-up

This was set at 6 weeks (as per previous studies; some studies had short intervals of up to 7 days immediately post therapy) [5, 9, 15, 18, 20, 26, 27]. Follow-up interviews were conducted via Physical review or telephone interview (has been validated in feasibility studies [5, 9, 15, 18, 20, 26, 27] and conducted. The follow-up interviews allowed the participants in the intervention arm (Group 1) to review the typed legacy document depicting the previous session on Dignity therapy and thereafter fill the ESAS form. The control arm filled the ESAS form as well.

Patients who were lost to follow up or died in the course of the study were included in the analysis as per the intention to treat principle.

### Data analysis

Categorical data was presented as frequencies and percentages while continuous data was presented as mean and standard deviations. Normality tests were done on the continuous data using Shapiro Wik tests. Exploratory subgroup analysis was performed for gender and age groups (below 45 years and above 45 years). Frequency distributions for types of cancers and comorbidities by age and gender were presented in tables and Charts.

The primary outcome was measured as summated and symptom specific ESAS scores at follow up. Symptom specific scores range from 0 to 10, with lower scores signifying better symptom outcomes. The Summated scores were calculated by adding scores on each ESAS item to generate one score out of 100.

To adjust for possible imbalances in baseline ESAS measures, multivariate analysis i.e. a general linear model (GLM) was conducted to determine a statistically significant difference between the intervention and control groups (fixed factors) on mean symptom distress score while controlling for baseline ESAS scores (continuous covariate).

## Results

### Participant flow

**Fig. 1:**
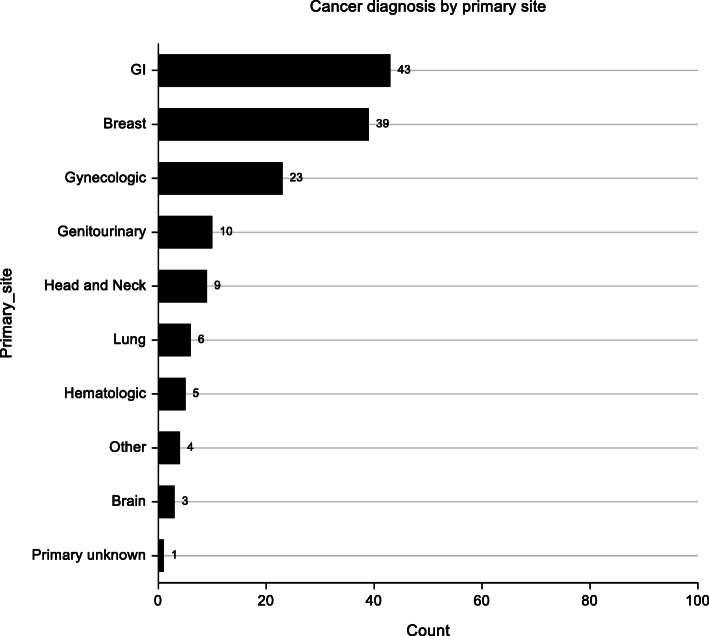
Bar graph illustrating distribution of malignancies among study participants.

Participants were recruited between August 2016 and March 2017. About 205 patients were assessed for eligibility, out of which 61 did not meet the eligibility criteria. Reasons for ineligibility included ongoing acute illness (*n* = 6), predominant communication in Kiswahili (Kenya’s official language) (*n* = 38), refusal to participate in the study (*n* = 17) and 9 who were acutely ill. Of the remaining 144 that were eligible, 72 were randomized to the intervention group, and 72 to the control group. Of the ones randomized, five were lost to follow-up in the Dignity therapy group while two died before post assessment. In the usual care group, six were lost to follow-up and five died before post assessment. Only 126 patients completed the study. One hundred and fourty three participants (one was excluded due to repetitive enrollment) were included for primary analysis; 72 in the intervention group, and 72 in the control group. We included the ones who died or were lost to follow-up as per the intention to treat principle, with one excluded due to repetition. Recruitment ended once the target number of patients was reached (Fig. 1). Follow-up assessments were conducted after 6 weeks after which trial was concluded.

### Baseline clinical and demographic characteristics

**Fig. 2:**
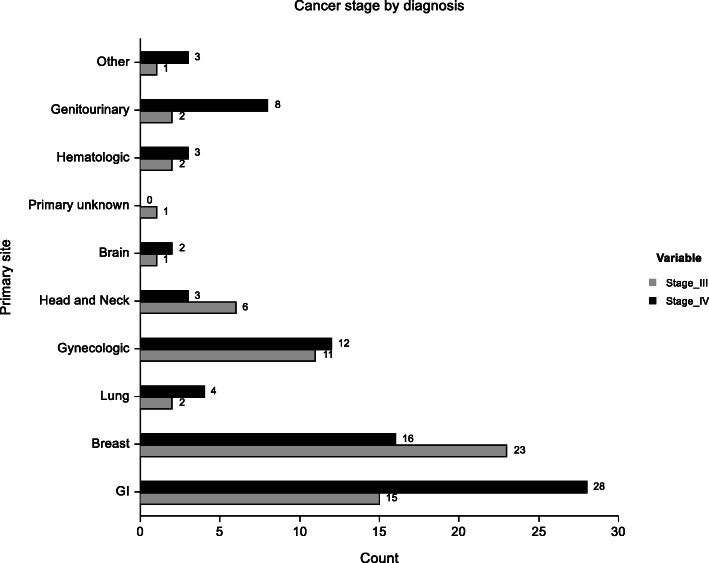
Bar graph illustrating staging of different malignancies among study participants.

**Fig. 3:**
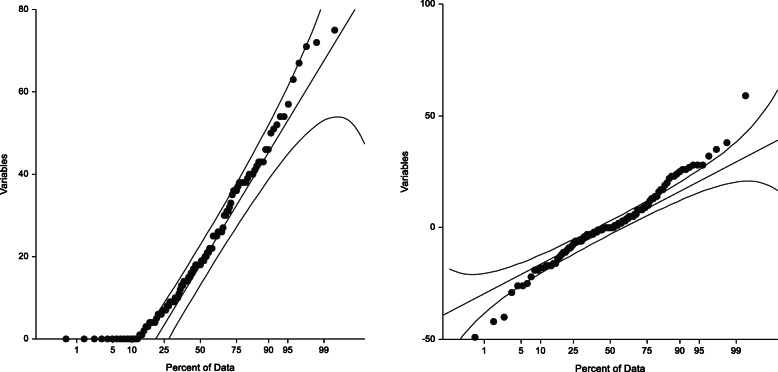
Are probability plots for mean follow up scores and the mean difference in follow up scores between intervention and control groups respectively.

A descriptive comparison of intervention and control groups did not reveal differences with regard to clinical or demographic characteristics. The mean age of participants was 50.5 and 52.5 years in the intervention and control group respectively. More females participated in the study compared to men in both groups. Breast cancer was the most common in both groups while stage IV disease was the most prevalent in both groups receiving palliative care. The most common form of treatment as per the time of participant recruitment was chemotherapy followed by radiotherapy, combined radiotherapy and chemotherapy with surgery and hormonal treatment having the lowest number of patients. A graphical representation is shown in Figs. 2 and 3.

### Primary outcomes

The Student t-test was utilized first before adjusted analysis using the general linear model (GLM). The primary analysis results showed higher scores for the DT group (change in mean = 1.57) compared to the UC group (change in mean = − 0.74) yielding a non-statistically significant difference in change scores of 1.44 (*p* = 0.670; 95% CI − 5.20 to 8.06). Therefore, dignity therapy incorporated in routine clinical care was not shown to statistically improve the quality of life of these patients as compared to routine care only.

Generalized linear models were used to adjust the baseline scores in order to find associations in mean scores at 6 weeks follow up. After adjusting the baseline scores, the results showed no statistically significance in summated post assessment scores (*p* = 0.127).

#### Symptom specific analysis

An independent student’s t-test evaluating symptoms in the two groups at baseline and at 6 weeks was conducted. Statistically significant differences within individual groups were noted in anxiety (Dignity group-*p* = 0.005) and nausea (Control group, *p* = 0.051) in usual therapy group. This suggested that dignity therapy improved anxiety in the intervention group and patients receiving usual care had overall reductions in nausea (likely due to medications given by primary care giver).

## Discussion

**Fig. 4:**
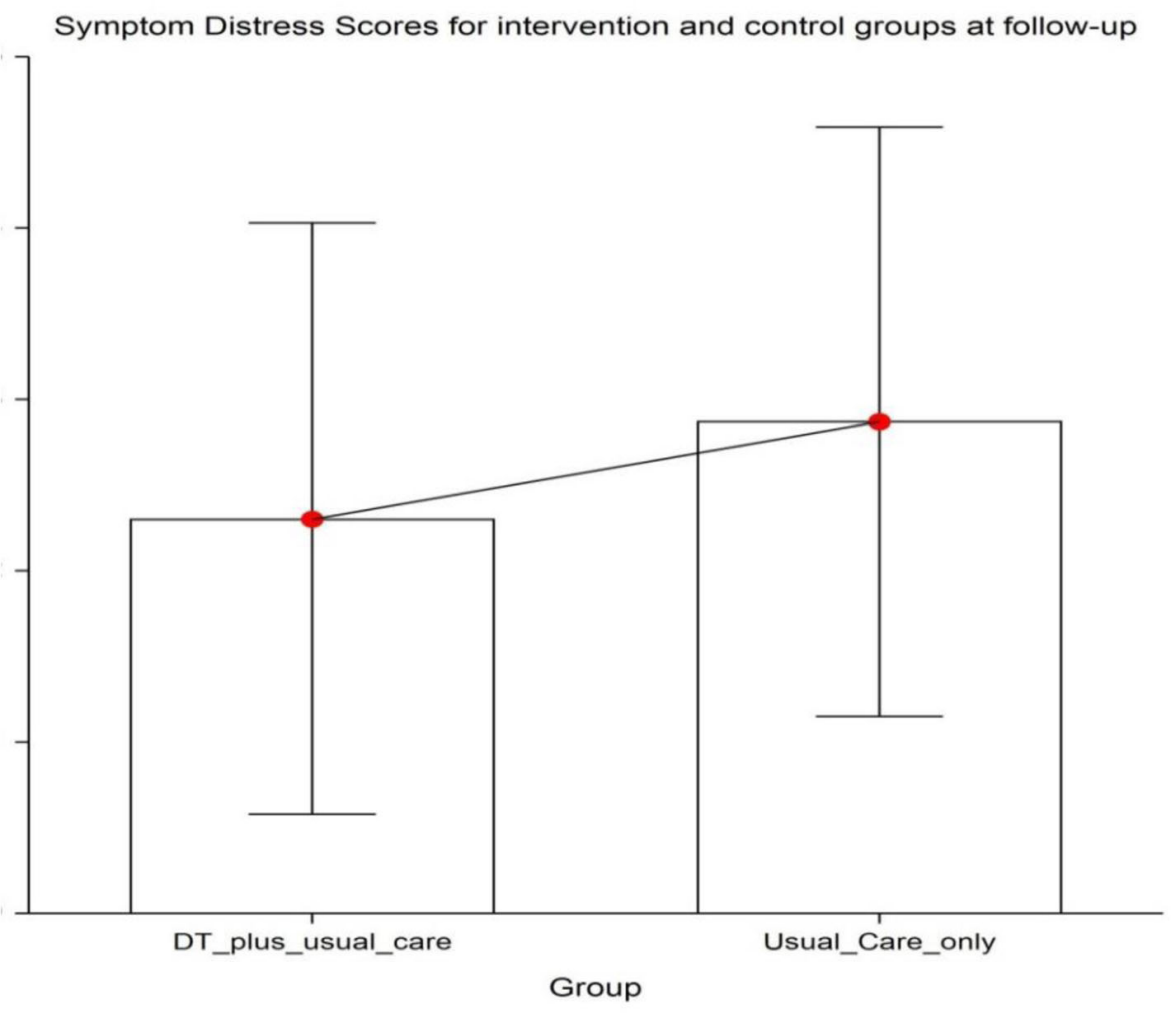
below: Box plot showing mean symptom distress scores for intervention group (18.18) and control group on follow-up (26.78).

This single center randomized controlled trial assessed the effect of dignity therapy on quality of life in advanced cancer patients. Dignity therapy in addition to routine clinical care was not shown to statistically improve the quality of life of these patients as compared to routine care only. There was symptom improvement in anxiety levels of the patients studied Fig. 4.

**Fig. 5:**
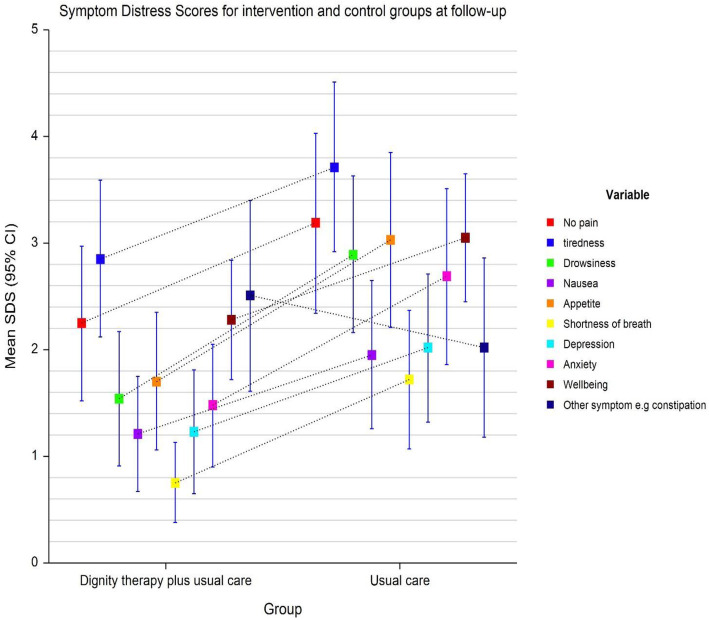
Symptom distress scores for intervention and control groups at followup.

**Fig. 6:**
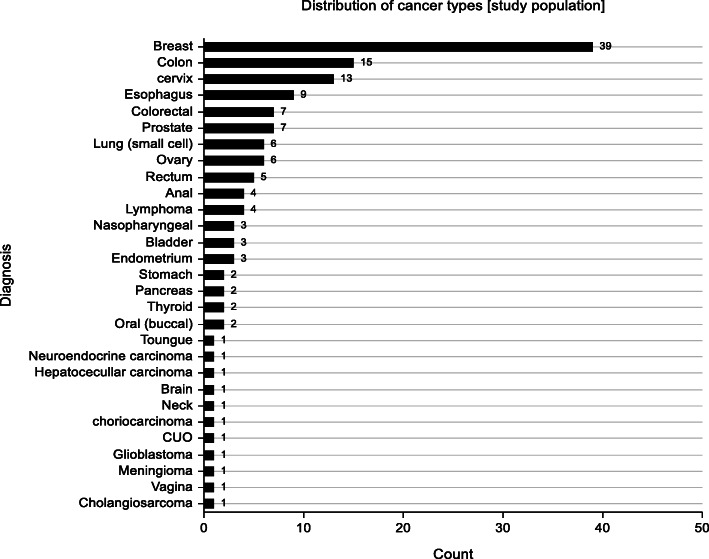
Frequency graph illustrating distribution of cancer types in study population.

**Fig. 7:**
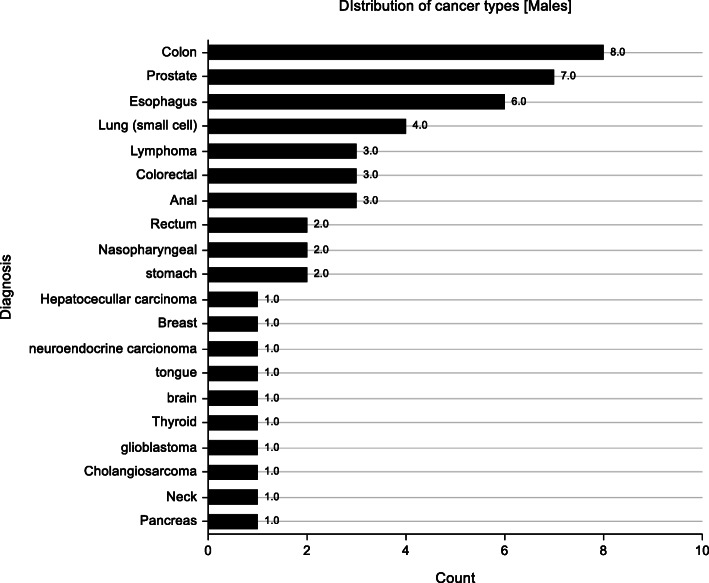
Frequency graph illustrating distribution of cancer types in Male population.

Palliative care in advanced cancer patients i.e. stage 3 or 4, aside from relieving distressing physical symptoms is aimed at improving quality of life. Quality of life is a multi-dimensional concept that looks at a patient holistically i.e. physical, psychological and spiritual aspects. Few RCT’s to date have been conducted on DT. Previous studies on DT focused on relieving distress symptoms as well as anxiety and depression among other symptoms (Fig. 5). Only one assessed quality of life as a primary outcome and even then no marked improvement was seen [9]. This study showed improved anxiety symptoms with a trend towards significance (*p* = 0.059) in the DT group and this mirrors the study done by Juliao et al. that showed that DT was associated with lower depression scores at up to 2 weeks of follow-up (*p* < 0.0001) and lower anxiety (*P* < 0.0001) within 3 weeks [16, 17]. The study done by Juliao et al. in 2014 is quite similar to this study with subtle differences. In similarity, both studies had a similar population with a mean age of 66.1 in Juliao’s study compared to 51.8 in this study. Majority of the population were female cancer patients on both studies (Fig. 6). Also similar is the fact that the primary investigators were involved in both studies and sessions lasted an average of 30 min. A stark difference in the studies is that Juliao explored other sociodemographic characteristics which this study did not e.g. race, marital status, religion and education. In the Juliao et al. study, it was found that these patients had high depression and anxiety scores even at baseline and demonstrated the positive effect of dignity therapy on these symptoms [16, 17]. The study showed that the two symptoms were reduced significantly with DT as an intervention. This AKUHN study also explored baseline anxiety scores and found that though not marked, they were significantly improved by Dignity therapy (*p* = 0.059) showing a trend towards significance. The choice to use the ESAS as a tool was its simplicity as shown in studies with similar designs e.g. Juliao et al (Fig. 7).

**Fig. 8:**
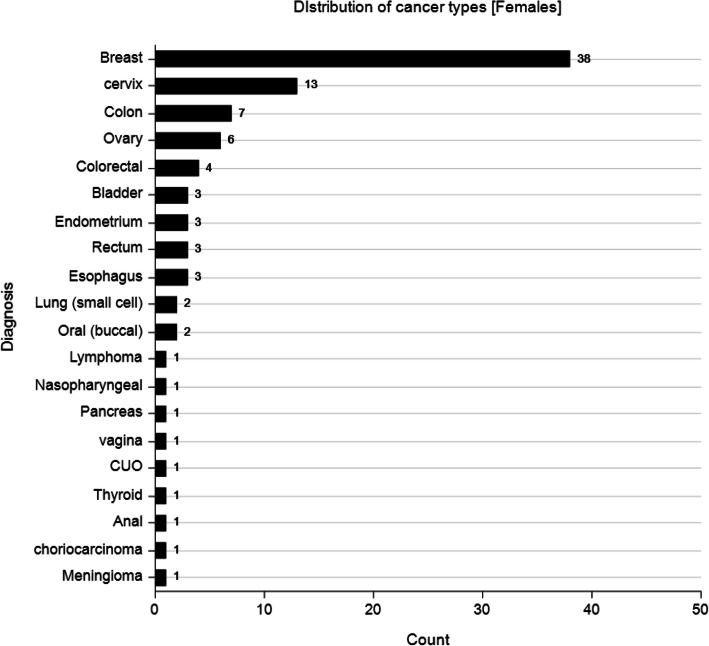
Frequency graph illustrating distribution of cancer types in female population

**Fig. 9:**
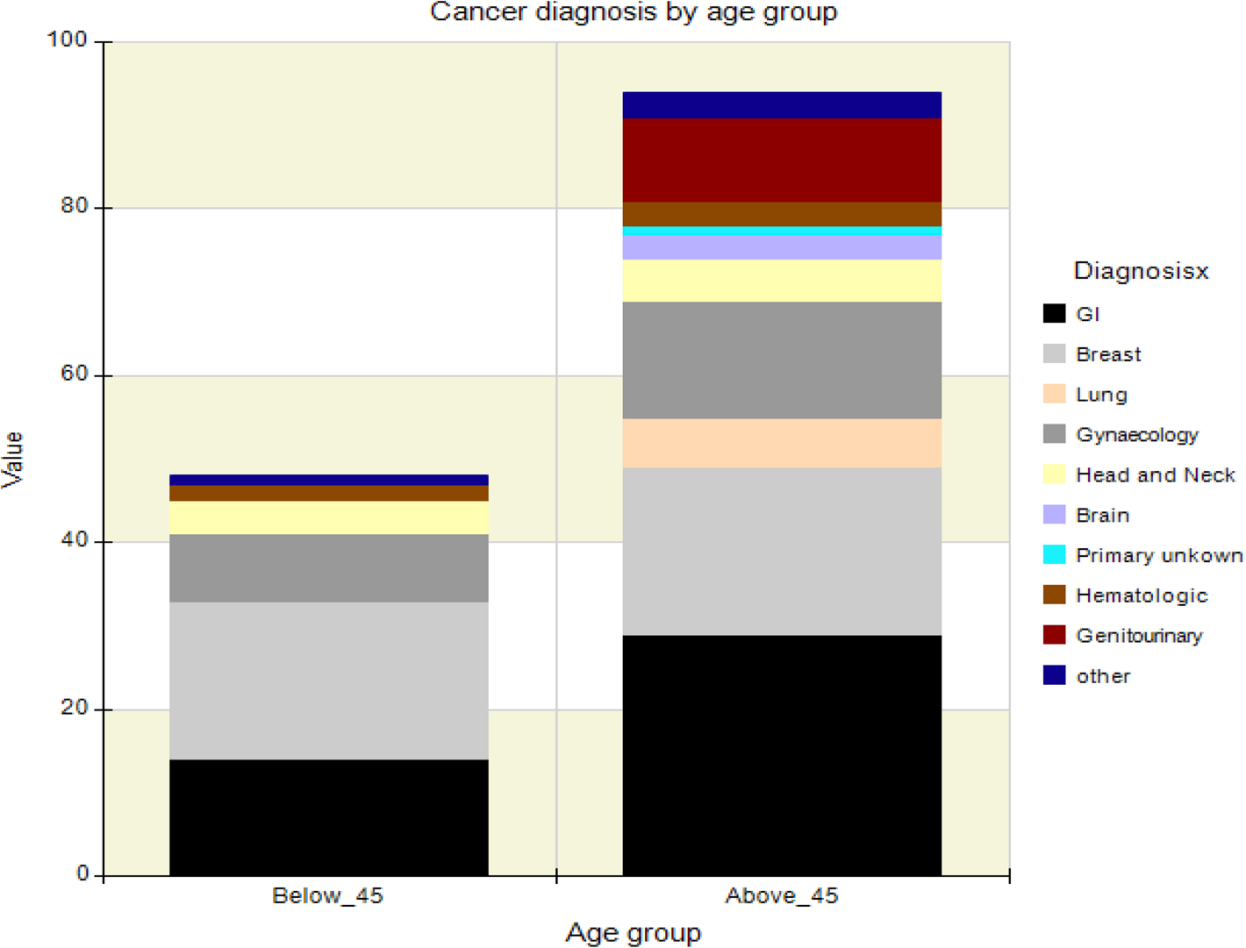
Bar graph illustrating distribution of cancer types according to age.

The effect on depression and anxiety is expected due to the nature of the illness, which results in increased anxiety levels and depression. This has been shown in numerous studies [16, 17] more so in patients with advanced cancers. Patients nearing the eminent end of their lives have many needs in addition to physical ones. Psychological disturbances e.g. anxiety, poor social, emotional and cognitive function have been shown to profoundly affect such patients more than the physical symptoms. Treatment of anxiety helps alleviate these distressing symptoms and this in turn improves quality of life of palliative care patients [16, 17, 28, 29]. Perhaps dignity therapy provides a means that patients can address existential issues like unfinished business and assists them express their long-term desires hence manifesting as a reduction of anxiety. This is however merely a speculation and further studies are needed in this area (Figs. 8 and 9).

**Fig. 10:**
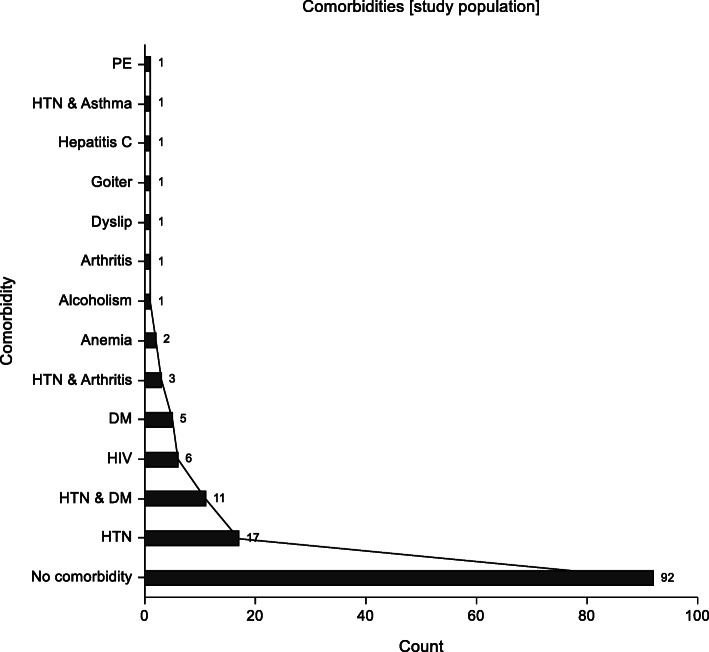
Frequency graph illustrating distribution of comorbidities in study population.

**Fig. 11:**
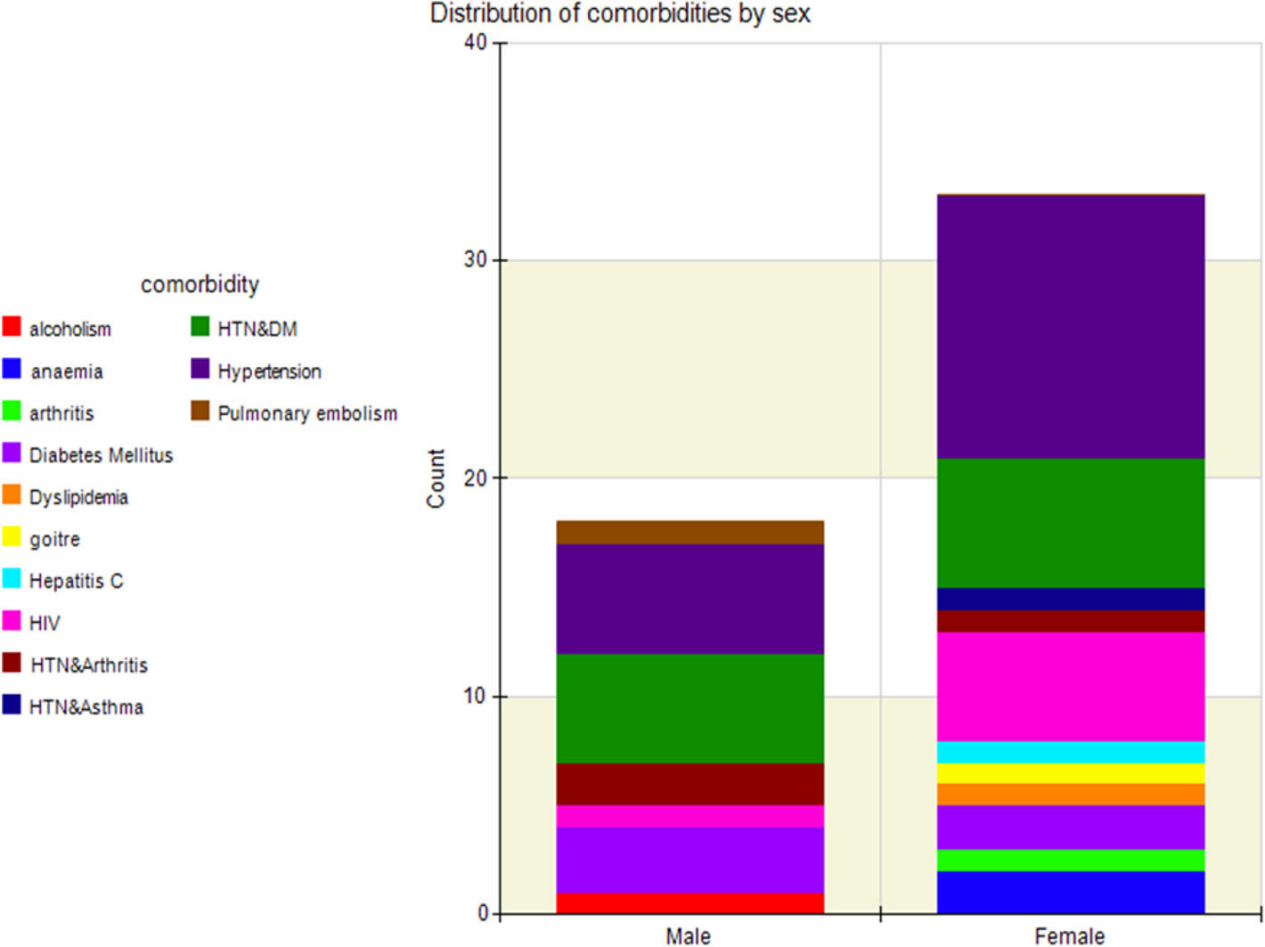
Bar graph illustrating distribution of comorbidities according to sex.

With the above, this study shows certain strengths including the fact that it is one of two, which attempted to evaluate effect of DT on quality of life. It is also the only study done in the subject in Africa thus far. This is important since palliative care is a neglected area in treatment of advanced cancer patients in Kenya. The need to incorporate it into clinical care is aligned with the need of dignity therapy to improve quality of life, more so in reducing anxiety surrounding advanced cancer management (Figs. 10 and 11).

Limitations to this study have been recognized including the fact that this study was conducted solely at a private institution and is not a reflection of the wider scope of healthcare in Kenya, which is mostly rural, and resource poor hence may not be applicable these set-ups. A second limitation is the possibility that this study may have been underpowered hence not showing statistical significance in the primary outcome. Of note, the biggest study to date [9] had a sample size of 441 while this study had 144. This study mirrored the smaller studies done [12–15, 18–21, 30, 31] which showed a similar limitation of under powering.

Another area of bias in the study is the use of single blinding which was a significant limitation.

The third limitation is the short time taken to do the study in most patients i.e. 30-60 min minutes. The counsellors undertaking the study took about 10 min to establish rapport between the patients. During this time, pre-assessment ESAS forms were filled. Could we have detected a statistical significance in scores had the sessions lasted longer and thus had a larger impact? Perhaps. This is however controversial since we have seen from the results that even the limited time given for dignity therapy had a profound effect on Anxiety.

The fourth limitation encountered is in data collection. Data was mainly from the medical oncology and surgical oncology clinics. Gynaecology clinics were few. This could have skewed the results especially in representation of the common malignancies. Only three persons conducted the intervention (Dignity therapy) i.e. the principal investigator and two professional counsellors. It was difficult to recruit other professionals due to limited funds and strict institutional policies. More personnel are needed in future studies. In addition, we do recognise that different counsellors approach subject matter differently; this could have been a major negative influence in the study since standardisation of counselling technique is difficult.

It is also important to note that the ESAS-r document though validated in the west has not been validated in our population. This could have limited its use in our indigenous population though patients accessing AKUHN are cosmopolitan.

Stigma facing the terminally ill patients was also an important limitation in this study. Many patients declined involvement due to fear of stigma. Many who went through the therapy reported feeling as though it was a “death sentence”. Patients need to be educated on the benefits of dignity therapy and this should be instituted early in their care.

Lastly, this Study focused on some aspects of quality of life, which comprised of nine symptoms from the ESAS. It did not assess other aspects e.g. financial aspects, distress and dying concepts, hope, boredom, performance etc. Future studies need to be done to incorporate the above.

## Conclusion

Despite having had no statistical effect on the overall quality of life score, there was clinical improvement in follow-up summated symptom scores as from some previous studies [23, 24]. This improvement in summated symptom score in the DT group compared to the control group is a good indicator of quality of life. In addition, symptom scores of anxiety showed a trend towards significance *p* = 0.059.

Therefore, incorporating dignity therapy in clinical care more so in palliative patients (especially those with anxiety) with advanced cancers may be beneficial. Due to the limitation of sample size and under powering, a larger study should be conducted on this subject. Further studies should also incorporate the patients’ socio-demographic factor so as to capture a larger model of quality of life.

## Supplementary information

**Additional file 1.**

## Data Availability

The datasets used and/or analyzed during the current study are available from the corresponding author on reasonable request. In addition, trial data can be accessed from the Pan African Clinical trials site, registration number PACTR201604001447244.

## Abbreviations

AKUHN: Aga khan University Hospital, Nairobi
DT: Dignity therapy
ESAS: Edmonton symptom assessment scale
HRQoL: Health related quality of life
IT: Information Technology (Computerized systems)
KnH: Kenyatta National Hospital, Nairobi
Qc: Quality control
QoL: Quality of Life
SD: Standard deviation
SEM: Standard error of the mean
