# Supplementary material for: Randomized control trial of advanced cancer patients at a private hospital in Kenya and the impact of dignity therapy on quality of life
Source: BMC Palliat Care. 2020 Jul 23;19:114. doi: 10.1186/s12904-020-00614-0 (PMC7379366; doi:10.1186/s12904-020-00614-0)
Supplement: Supplementary file 1 — Additional file 1. [file 12904_2020_614_MOESM1_ESM.docx]

**APPENDICES**

Appendix I EXPLANATION FORM

**EFFECT OF TARGETED PSYCHOTHERAPY (DIGNITY THERAPY) on QUALITY OF LIFE IN PALLIATIVE CARE PATIENTS: a parallel group randomized control trial.**

Name of principal investigator: Dr. Miriam W. Gatehi

Name of the institution: Aga Khan University Hospital, Nairobi

**Introduction**

As indicated above, my name is Dr. Miriam Gatehi, a doctor training for her postgraduate medical education at the Aga khan University Hospital.

This document gives a detailed account of the research that I am undertaking involving patients such as you.

The research is on the effects of a form of psychological therapy called dignity therapy on your quality of life.

I will give you information about the research and invite you to participate.

Before conducting the above, I will explain it to you in detail and you are encouraged to ask questions freely in areas where you may not understand.

**Purpose of the research**

To guide clinical care in assessing your current quality of life so as to intervene and dealing with any underlying issue in terms of physical and psychological wellbeing. Those selected to do so will receive a form of counselling known as dignity therapy.

**Type of research intervention**

We would like to assess the effect of this form of counselling i.e. dignity therapy on your quality of life.

NB: You will be required to undergo balloting in order to receive this form of counselling. Those who do not receive the counselling will still undergo a full symptom assessment and treated appropriately.

**Patient Selection**

You have been selected to take part in the study as a patient with advanced cancer i.e. stage 3 and 4. Although you are encouraged to participate, this process is entirely voluntary and you are free to decide whether you wish to take part in the study.

In case you decline to participate you will still be able to access health services here at the Aga Khan University Hospital.

If you decide to participate in the study, you are free to leave at any point during the process.

**Information on the Study**

If you agree to participate, you will have your details filled in terms of age, gender, diagnosis and stage and any other illness you may have coexisting.

Thereafter you will be required to sign a consent that you have agreed to participate.

A balloting will be done where you will be split into two groups. G1 and G2. Both groups will be required to fill a form before beginning the experiment i.e. Edmonton symptom assessment scale.

Depending on the group chosen, you will receive a 15-20-minute counselling session that seeks to address your life history by focusing on happy memories and whose aim is to improve your quality of life. This will be written down and presented to you for safekeeping. You are free to give it to any of your loved ones.

Thereafter you are required to review it at least once before your next visit where you will be required to fill some form after review. If you forget to do so, a copy will be provided at your next visit or dictated to you via telephone interview. If by telephone interview, you will direct the person calling (primary investigator) how you wish to fill the forms.

Alternatively, you may receive usual services (which may include counselling) and you will be required to fill the forms on review, which is set at 2 weeks to 1 month after the initial visit.

**Duration**

The research will take place during your routine visit to your doctor and a follow up 6 weeks thereafter.

**Risks**

There are no documented risks (psychological or otherwise) to this research. However, should you develop any distressing symptom you will be treated appropriately by our counsellors or designated doctors within the institution.

Benefits of conducting this research will include provision of knowledge to the medical team on how best to improve the quality of life of patients such as yourself. It will also help us determine whether there is a benefit in this form of counselling i.e. dignity therapy on your quality of life as compared to no counselling at all.

This will help us improve your care in future.

**Compensation**

Study participants will NOT BE COMPENSATED. You are also not required to pay for this service. This is separate from your usual reviews here which will be charged.

**Confidentiality**

As a participant in the study, you are guaranteed full confidentiality.

Your name and personal contacts will only appear in my records and not on the study documents, (these are coded). Only persons involved in the study shall have access to your information.

Feel free to address any concern you may have using the contacts below;

**Dr Miriam W. Gatehi**

**Tel number 0722441891**

**P.O Box 30270-00100**

**Aga Khan University Hospital, Nairobi, Kenya**

Appendix II: CONSENT FORM

I…...........................................................................................................hereby agree to take part in this study, having been fully informed of the nature of the study by Dr Gatehi.M.W.

Date…………………………………………….

Signature……………………………………….

I, Dr. Gatehi. M. W, has fully explained to the patient the nature of the study and hereby undersign

Date………………………………………………Signature………………………………………

Appendix III: REVISED EDMUNTON SYMPTOM AND ASSESSMENT SCALE
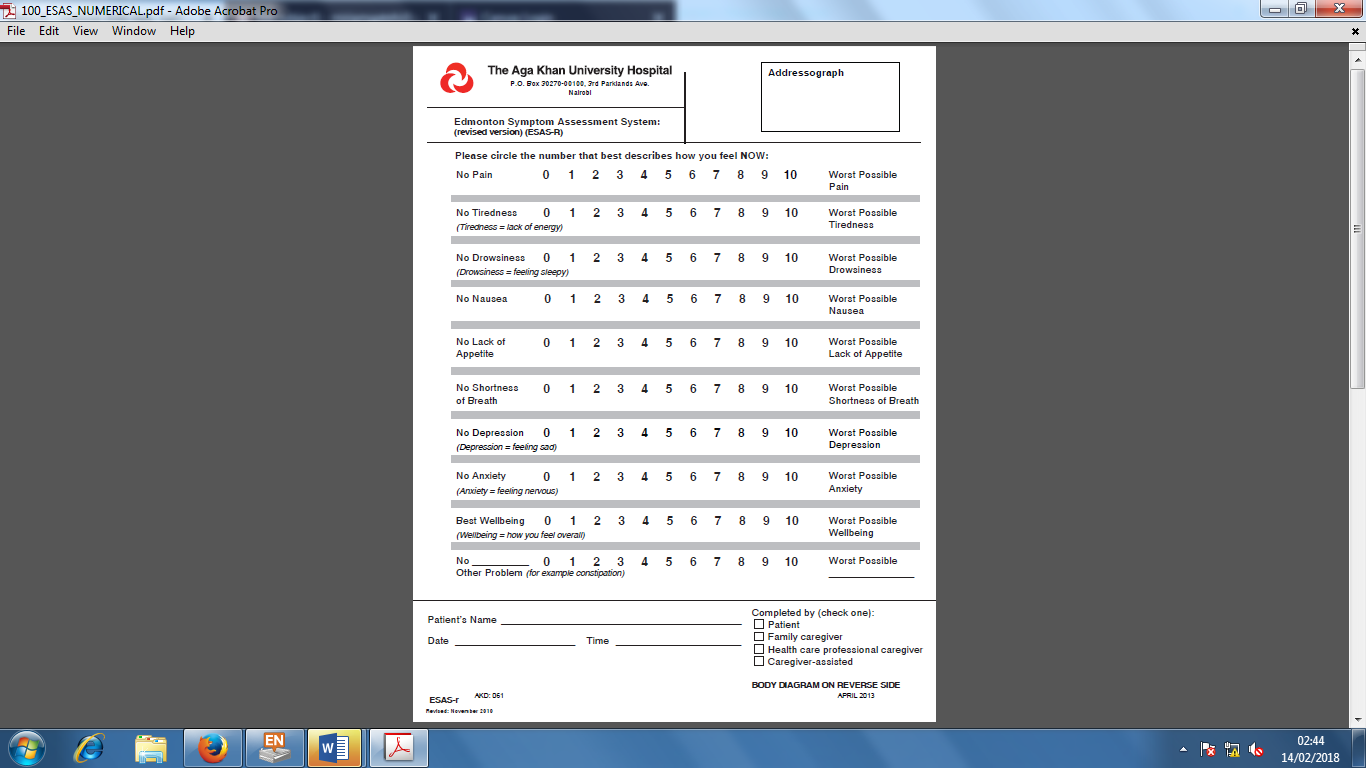


Appendix IV: DIGNITY THERAPY MODEL

**
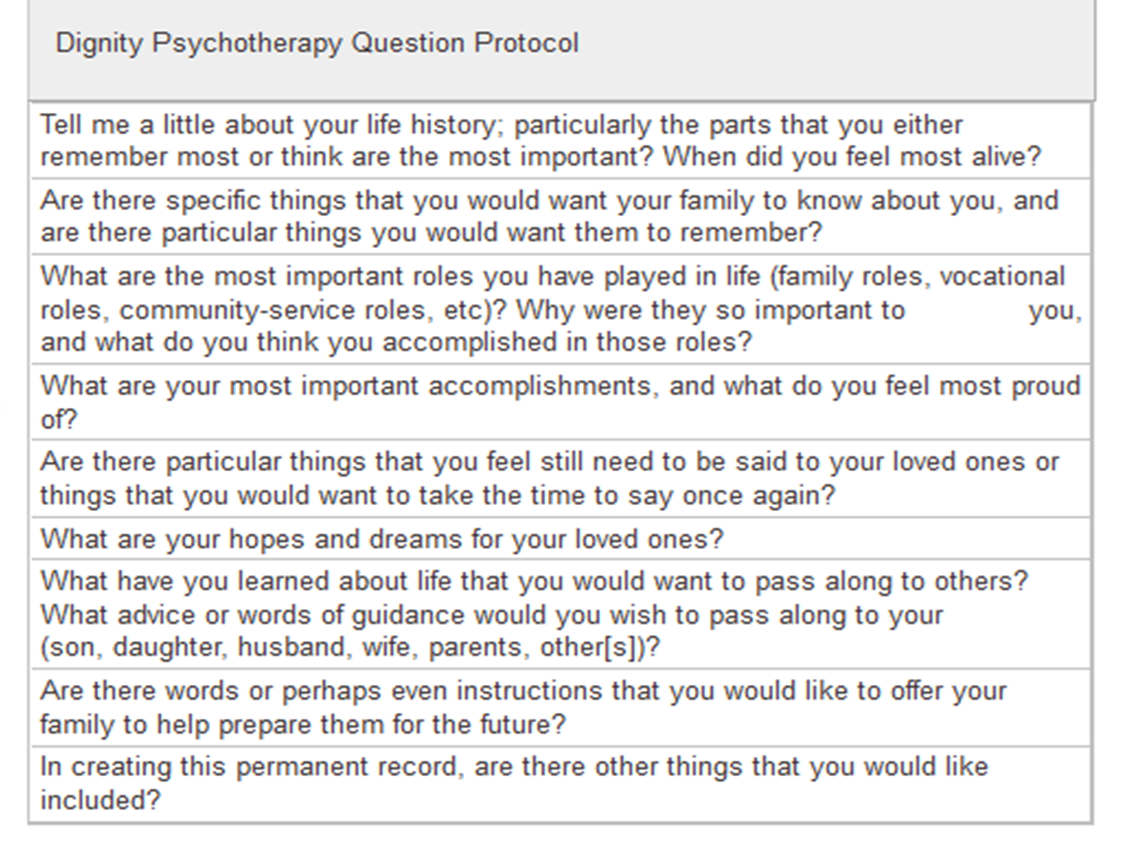
**

Appendix V: ADDITIONAL FILES

Supplementary Material 1: Consort 2010 diagram depicting guidelines in reporting randomized trials and the input from the study.
